# Chemotherapy with cisplatin and vinorelbine for elderly patients with locally advanced or metastatic non-small-cell lung cancer (NSCLC)

**DOI:** 10.1186/1471-2407-4-69

**Published:** 2004-09-29

**Authors:** José Rodrigues Pereira, Sandro J Martins, Sueli M Nikaedo, Flora K Ikari

**Affiliations:** 1 Lung Cancer Division, Instituto do Câncer Arnaldo Vieira de Carvalho, R. Martinico Prado 26 cj. 101, 01224-010 São Paulo, Brazil; 2 Pulmonary Division, University of São Paulo Medical School, Av. Dr. Arnaldo 455, 01246-903 São Paulo, Brazil; 3 Current address: Oncology Unit, Hospital Santa Izabel, Pca Almeida Couto 500, 40050-410 Salvador, Brazil

## Abstract

**Background:**

Although modest improvements in the survival of patients with non-small cell lung cancer (NSCLC) can be achieved with cisplatin-based chemotherapy (CT), its value is disputed in the geriatric setting. In this study, we evaluate the feasibility of vinorelbine/cisplatin CT for elderly NSCLC patients.

**Methods:**

In this pilot phase I/II trial, all patients received CT with vinorelbine 25 mg/m^2^, on day 1 and 8, and cisplatin on day 1, in 28 days-cycles. After stratification for age (up to 75 years), younger patients were sequentially allocated to moderate cisplatin doses (80 mg/m^2 ^or 90 mg/m^2^), and older patients were allocated to lower cisplatin doses (60 mg/m^2 ^or 70 mg/m^2^). We recruited patients aged over 70 years with newly diagnosed NSCLC, clinical stage III or IV, Karnofsky performance status ≥ 70%, normal serum creatinine, peripheral neuropathy ≤ grade 1, and no prior cancer therapy.

**Results:**

Analysis was by intention to treat. Main toxicities (grade 3–4) was as follows: neutropenia, 20%; anemia, 11%; and thrombocytopenia, 2%; alopecia, 55%; fatigue, 11%; and peripheral neurotoxicity, 2%. No grade 3–4 emesis or renal toxicity occurred. Global median time to progression (TTP) and overall survival (OS) were 27.0 (95% CI: 10.1 to 43.7) weeks and 30.1 (95% CI: 24.4 to 35.8) weeks; 1- and 2-year survival rates were 36.3% and 13.2%, respectively. Overall response rate was 50.0% (95% CI: 35.4% to 64.5%), with 1 complete response; no difference on response rate was noticed according to cisplatin dose. Median overall survival was 30.1 weeks, with 1- and 2-year survival rates of 36.3% and 13.2%, respectively.

**Conclusion:**

Age does not preclude assessment on the role of cisplatin-vinorelbine CT for elderly NSCLC patients with good performance status and adequate bodily functions.

## Background

Lung cancer is a disease with a great incidence in older people. In Brazil, its incidence rate in male sex was expected to reach 18.8:100.000 in 1999, and aged patients may have accounted for 57% of all lung cancer deaths. Unfortunately, up to two thirds will present at diagnosis with advanced disease, requiring chemotherapy (CT) [1].

Vinorelbine, a semi synthetic vinca alkaloid, is a highly active drug for NSCLC and its association with cisplatin is worthwhile. European and Southwest Oncology Group trials demonstrated that vinorelbine/cisplatin (VP) offer therapeutic advantage over both drugs alone [2-4], previous cisplatin-based schedules [3]; comparing to taxane combinations, VP is therapeutically equivalent to carboplatin/paclitaxel [5] or carboplatin/docetaxel [6] but inferior to cisplatin/docetaxel [6].

Although modest improvements in the survival of patients with non-small cell lung cancer (NSCLC) can be achieved with cisplatin-based CT [7], its value is disputed in the geriatric context. The simultaneous presence of several diseases and homeostenosis, an age-related physiologic process that change the way that the body handles drugs, can shift therapeutic index, allowing harm outweigh any survival gain. On the other hand, underdiagnosis and undertreatment of lung cancer in the elderly is a fact, often explained in terms of ageism in medical oncology staff [8] and people's beliefs and fears about the disease and its treatment [8,9]. Whether we should treat or not aged patients with cisplatin-based CT surely is an unsolved issue. In this study, we evaluated the feasibleness and activity of vinorelbine/cisplatin CT for elderly NSCLC patients.

## Methods

This study enrolled patients older than 70 years with unresectable locally advanced or metastatic NSCLC. Informed consent was obtained from patients and their relatives, as approved by the Institutional Ethical Committee. Inclusion criteria: all patients had to have histologically confirmed NSCLC; Karnofsky performance status ≥ 70; measurable disease; adequate bone marrow reserve (neutrophils ≥ 2 × 10^9^/L and platelets ≥ 100 × 10^9^/L), bilirubin under 1.25 times upper normal value (UNV), aspartate aminotransferase/alanine aminotransferase (AST/ALT) under 2 times UNV, and renal function (creatinine level under 120 μmol/L); no symptomatic brain metastasis; no prior cancer therapy; no indication for palliative radiation therapy; no previous or concomitant malignancy; and adequate social support. Exclusion criteria were symptomatic peripheral neuropathy and comorbidity regarded as an impediment for CT, such as renal disease, heart failure, coronary heart disease, uncontrolled infection, and cognitive impairment.

Baseline work up included a medical history and physical examination; whole blood count (WBC) and biochemistry; chest x-ray; bone scintigraphy scan; chest, abdominal and brain computed axial tomography scans; and electrocardiogram. Although pretreatment bone scan and brain CAT scan are recommended only when signs or symptoms of disease are present, they were added here due to Institutional Protocol Reviewers recommendation.

Treatment consisted of vinorelbine 25 mg/m^2 ^on days 1 and 8, administered intravenously in bolus, followed by intravenous cisplatin over 1 hour. CT was administered every 4 weeks. Prophylactic anti-emetic drugs (intra venous dexamethasone 20 mg and ondansetron 16 mg) and fluid hydration (0.9% saline, 1 L/m^2^; and magnesium sulfate, 20 mmol) was used to minimize renal toxicity. Dexamethasone 4 mg PO BID plus metoclopramide 10 mg PO QID for 4 days was used to prevent delayed emesis. Patients were time-sequentially assigned to one of two groups, from lower to higher doses, according to age strata. Study doses were defined based on previous reports on renal tolerance of cisplatin in geriatric patients [10,11]. Age stratification was arbitrary. Those aged up to 75 years received cisplatin 60 or 70 mg/m^2 ^and those aged 70 to 75 received cisplatin 80 or 90 mg/m^2^. Assignation to high dose groups (70 mg/m^2 ^and 90 mg/m^2^) occurred after evaluation of toxicity at inferior doses, evaluated according to National Cancer Institute criteria. The protocol required a minimum of 6 and a maximum of 18 patients per group after the 1st cycle for safety analysis.

Chemotherapy doses were reduced for haematological, neurological, hepatic and renal toxicities. Toxicity was graded according to National Cancer Institute (NCI) common toxicity criteria guidelines. Changes in dosage were based on WBC results obtained on day 1 of treatment; if neutrophils were <1.5 × 10^9^/L and platelets were <100 × 10^9^/L, treatment was delayed by 1 week. Treament on days 8 had to be cancelled if neutrophil counts were <1.0 × 10^9^/L and platelets were <100 × 10^9^/L. If treatment could not be given after a 2-week interval because of haematological toxicity, it had to be discontinued and the patient withdrawn from the study. Concomitant use of hematopoietic growth factors were not allowed in the first treatment cycle but were administered subsequently on individual basis. Neurological toxicity above grade 2 resulted in suspension of treatment; ototoxicity grade 2 or 3 resulted in a 50% dose reduction of cisplatin.

The following dose modifications of vinorelbine were set based on AST/ALT (aspartate aminotransferase/alanine aminotransferase) and bilirubin values on day 1 or day 8 of treatment: if AST/ALT were between 5.1 and 20.0 × UNV or bilirubin was between 1.5 and 3.0 × UNV, dosing was cancelled and the patient was reassessed 1 week later. If AST/ALT were >20.0 × UNV or bilirubin was >3.0 × UNV, vinorelbine was discontinued. If serum creatinine was grade >1, the dose was delayed by 1 week and the test repeated. After a 2-week delay, the patient was taken off the study. WBC and biochemistry were also performed on day + 14 of treatment.

We intended to administer a maximum of four CT cycles followed by radiation therapy in responding patients with stage III disease and six CT cycles in patients with wet IIIB or stage IV disease. Notwithstanding radical radiation therapy (RT) should deliver a total dose down to 66 Gy, covering tumor site and regional lymph nodes, and palliative therapy could use doses under to 45 Gy, the final choice of dose, fractioning, irradiated volume, and energies of radiation was at the radiation oncologist's discretion. Treatment interruption was allowed in case of disease progression, severe adverse events, or patient preference.

Chest x-ray was performed before each cycle, and CAT scans every two cycle for response evaluation. Tumor response was recorded according to World Health Organization criteria and measured by the same observer (JRP). All responses had to be confirmed 3–4 weeks from initial evaluation. We reported here the best response designation recorded from the start of treatment until disease progression. Patients stopping treatment with an unconfirmed response, or only short stabilisation were considered as inevaluable, unless the response or stabilisation was further confirmed in the absence of any treatment. Patients were monitored for the first month off-study then followed up every 2–3 months.

The dose intensity was calculated for both drugs by dividing the actual dose delivered by the length of therapy. Toxic death was defined as death occurring during the chemotherapeutic phase (including four weeks after its end) and due to drug toxicity. Early death was defined as death within four weeks after a chemotherapy cycle without severe toxicity and not related to the malignant disease. Response and survival were calculated by intention-to-treat. Progression was defined in relation to the best response obtained. The time to tumor progression lasted from the first day of treatment to the date of the first observation of progressive disease. Survival was defined as the time elapsed from the beginning of CT until death or last follow-up visit. Time-to-event analysis was performed using the Kaplan-Meier product-limit estimator. All analyses were carried out using a computer program (SPSS version 8.0, Chicago, USA).

## Results

Forty-four patients were recruited from July 1996 to June 1998; twenty-nine aged 70–75 year and fifteen older than 75 years. Cisplatin doses were as follows: in the older cohort, seven patients received 60 mg/m^2 ^and eight received 70 mg/m^2^; in the former, fifteen patients received 80 mg/m^2 ^and fourteen received 90 mg/m^2^. Patient characteristics are given in Table 1. Most of the patients presented stage III disease (56.8%) and squamous cell carcinoma (52.3%).

Treatment results are shown in Table 2. A total of 125 CT cycles were administered and the median was 3 (range: 1 to 6). No difference was noticed on the dose intensity achieved across the four groups (Kruskal-Wallis test, p = 0.13). Objective response could not be evaluated in six patients due to treatment discontinuation before cycle 2: early death (1), withdrawal of consent (2) and toxicity (3). Twelve out of 25 patients with stage III disease responded to CT and received radical radiation therapy (median delivered dose: 50 Gy; range: 40 Gy to 66 Gy). Fifteen patients (34.0%) received maximum allowed CT cycles; excessive toxicity (n = 8), progressive disease (n = 3), progressive disease after initial response (n = 13), and patient choice (n = 5) were reasons for protocol withdrawal. At a median follow-up time of 77.2 weeks, six patients were alive. Response rate (RR) was 50.0% (95% CI: 35.4% to 64.5%), with 1 complete response. Global median time to progression (TTP) and overall survival (OS) were 27.0 (95% CI: 10.1 to 43.7) weeks and 30.1 (95% CI: 24.4 to 35.8) weeks; 1- and 2-year survival rates were 36.3% and 13.2%, respectively. No significant difference was noticed in RR (p = 0.65), TTP (p = 0.62), and OS (p = 0.44) across study groups. There was no difference according to stage (III vs. IV) in RR (48% vs. 53%, p = 0.76), TTP (32.6 vs. 25.0 weeks, p = 0.73), or OS (31.7 vs. 28.6 weeks, p = 0.33). Likewise there was no difference according to age groups (70–74 vs. ≥ 75 years) in RR (55% vs. 40%, p = 0.34), TTP (31.7 vs. 28.6 weeks, p = 0.33), or OS (30.1 vs. 31.7 weeks, p = 0.76).

Toxicity data are presented in Table 3. Hematological toxicity (grade 3–4) was as follows: neutropenia, 20%; anemia, 11%; and thrombocytopenia, 2%. Common nonhematologic grade 3–4 side effects were alopecia (18%) and fatigue (11%); severe peripheral neurotoxicity occurred in one patient; neither severe emesis nor renal toxicity was noticed. At the highest cisplatin dose (90 mg/m2) there were two early deaths and one toxic death due to neutropenic sepsis. No case of febrile neutropenia was noticed.

## Discussion

Treatment of elderly NSCLC patients with cisplatin-based regimens has been a less contentious matter nowadays but toxicity remains a major issue. In our pilot study, chemotherapy with cisplatin 70–80 mg/m^2 ^on day 1 plus vinorelbine 25 mg/m^2 ^on days 1 and 8, repeated each 28 days per in the maximum four cycles, was feasible for elderly NSCLC patients. Neurological and renal tolerance was particularly good. At the time we developed the protocol, no quality-of-life instrument had been validated for use in Brazil. Thus, a drawback in our study is the absence of quality-of-life analysis, which precludes evaluation of key dimensions in geriatric oncology.

Cisplatin induces a sensory neuropathy due to axonal damage that is dependent on the total-dose and single-dose intensity [12], and it is also time-dependent [13], making histological lesions more common than clinical toxicity. Although in this trial most patients received moderate cisplatin doses, only one had grade 3 peripheral neuropathy. Our data may reflect the low median of cycles actually administered rather than inaccuracy of clinical signs to evidence tissue lesion. Similarly, Ohe et al. [14] delivered a median of three cycle of cisplatin-containing CT and reported no case of severe neuropathy.

Cisplatin renal toxicity has been attributed to drug-protein interactions and the inactivation of specific brush border enzymes, resulting in damage of the loops of Henle, the distal tubules, and collecting ducts. Patients aged above 70 or even 80 are regarded as susceptible to cisplatin-induced renal damage as the younger counterparts [15,16] and current studies have reported a low incidence of renal toxicity in elderly patients [14,17]. No case of severe renal toxicity was noticed in our patients, as estimated by serum creatinine and its clearance (Cockroft-Gault method), but this finding may be artifactual due to small sample sizes, selection bias, and low sensitivity of estimated creatinine clearance to predict actual glomerular filtration rate (GFR) [18].

The observed response rate here was in the usually range of NSCLC phase II trials, but the absence of external review of radiological data and the widened confidence intervals expected because of small sample sizes in each group limit assertions that could otherwise be drawn. The 1-year survival rate (36%) was good but essentially equivalent to the reported elsewhere [19] for vinorelbine alone (32%) and inferior to the observed for weekly cisplatin-docetaxel (64%) [14]. Nonetheless, survival figures should be cautiously considered in this underpowered, heterogeneous, non-randomized pilot study.

Vinorelbine is a cytotoxic agent that clearly has expanded the therapeutic options for elderly NSCLC patients [20,21]. The next logical step to improve therapeutic indexes was to combine it with other active drugs. Recently, relevant results emerged from European phase III trials addressing the role of novel drugs in the treatment of elderly NSCLC patients [19,22-25]. The Elderly Lung Cancer Vinorelbine Italian Study (ELVIS) [19], interrupted for slow recruitment, evidenced an improvement of some lung cancer-related symptoms (pain and dyspnea), worsening of toxicity-related symptoms (cognitive function, constipation, and peripheral neuropathy), and a limited survival advantage (28 vs. 21 weeks) for single-agent vinorelbine as compared to supportive care, a survival gain that resembles the benefit reported by meta-analysis for nowadays considered substandard cisplatin-based regimens in advanced NSCLC.

Although sequential administration of drugs is an attractive option for aged or frail patients, a setting where minimal treatment-related toxicity should be pursued, research on the role of non-platinum combinations for elderly NSCLC patients aroused attention. The Southern Italy Cooperative Oncology Group (SICOG) evaluated whether the association of vinorelbine and gemcitabine would be better than vinorelbine alone. To date, final results of this trial have not to come. Despite an article focusing on the interim analysis of 120 patients (60 at each group) claimed a survival advantage for the combined arm [22], further intent-to-treat analysis including 18 patients more reached a less optimistic conclusion: median survival in the combined and single-agent arms were nearly the same (25 weeks and 23 weeks, respectively) and both values were deemed comparable to the observed in the supportive care arm of the ELVIS (21 weeks) [23]. Since that at least 152 patients was treated in the SICOG trial [24], a definite report of mature survival data is awaited. In addition, investigators of the Multicenter Italian Lung Cancer in the Elderly Study (MILES) [25] reported no survival benefit for the combination of vinorelbine plus gemcitabine in comparison to single-agent vinorelbine or gemcitabine in the treatment of elderly NSCLC patients.

The question whether or not cisplatin-containing regimens should be used to treat aged patients remains an important, still open, issue. As observed for paclitaxel-carboplatin [26], gemcitabine-cisplatin [27], and docetaxel-cisplatin [14] associations, the role of vinorelbine-cisplatin regimens deserve to be investigated. Until the outcome of large clinical trials addressing this issue proves at least the equivalence of newer drug associations to platinum-based regimens, as seems to be true for the combination of paclitaxel and gemcitabine [28], there are few reasons to preclude the evaluation of current combined regimens in the chemotherapy of elderly NSCLC patients with normal bodily functions and good performance status.

## Competing interests

This study was supported by Institutional funds only. Authors did hot received reimbursements, fees, funding, or salary from an organization that may in any way gain or lose financially from the publication of this paper in the past five years.

## Abbreviations (order of appearance)

NSCLC: non-small cell lung cancerCT: chemotherapy

NCI: National Cancer Institute

ELVIS: Elderly Lung Cancer Vinorelbine Italian Study

SICOG: Southern Italy Cooperative Oncology Group

GFR: glomerular filtration rate

## Authors' contributions

JRP was responsible for study conception, protocol conduction, and results interpretation. SJM carried out the data analysis and results discussion. SMN and FKI were responsible for patient care and data gathering

## Pre-publication history

The pre-publication history for this paper can be accessed here:



## Figures and Tables

**Table 1:** Patient characteristics

	Study Group				Total
		
	70 to 75 years		> 75 years		
			
	80 mg/m2 (n = 15)	90 mg/m2 (n = 14)	60 mg/m2 (n = 7)	70 mg/m2 (n = 8)	(n = 44)
Median age (range, in years)	73 (71 to 74)	73 (71 to 74)	79 (76 to 84)	77 (76 to 85)	74 (71 to 75)
Gender					
Male	12	12	5	5	34 (77%)
Female	3	2	2	3	10 (23%)
Karnofsky performance status					
70	8	7	4	3	22 (50%)
80 – 90	7	7	3	5	22 (50%)
Stage:					
IIIA	5	1	-	2	8 (18%)
IIIB	5	8	3	1	17 (39%)
IV	5	5	4	5	19 (43%)
Histology:					
Squamous cell carcinoma	9	5	2	7	23 (52%)
Adenocarcinoma	3	7	3	-	13 (30%)
Others	3	2	2	1	8 (18%)

**Table 2:** Therapy results

	Study Group				Total
		
	70 to 75 years		> 75 years		
			
	80 mg/m2 (n = 15)	90 mg/m2 (n = 14)	60 mg/m2 (n = 7)	70 mg/m2 (n = 8)	(n = 44)
Treatment administered					
Chemotherapy cycles					
No.	46	42	14	23	125
Median (range)	2 (1 to 4)	2 (1 to 5)	3 (1 to 5)	3 (1 to 6)	3 (1 to 6)
Vinorelbine (median, mg/m^2^/wk)	8.6	8.8	9.4	10.3	8.9
Cisplatin (median, mg/m^2^/wk)	13.6	15.9	11.2	14.4	14.5
Efficacy					
Complete response	-	-	-	1	1
Partial response	8	8	2	3	21
Stable disease	4	4	3	2	13
Progressive disease	2	1	-	-	3
Not evaluated	1	1	2	2	6
Overall response rate (%)	53	57	29	50	50
Time to progression (median, in weeks)	25.0	23.0	15.7	18.4	27.0
Overall Survival					
Median (weeks)	31.0	17.3	26.6	31.7	30.1
1-year (%)	40.0	35.7	28.5	37.5	36.3

**Table 3:** Patient tolerance

	70 to 75 years				> 75 years					
					
	80 mg/m2		90 mg/m2		60 mg/m2		70 mg/m2		Grade ≥ 3	
	3	4	3	4	3	4	3	4	n	%
Haematological										
Neutrophils	2	3	1	2	-	-	1	-	9	20
Haemoglobin	2	-	2	-	-	-	1	-	5	11
Platelets	-	-	-	-	-	-	1	-	1	2
Clinical										
Alopecia (grade 2)	(3)		(2)		(1)		(2)		8	18
Fatigue	1	-	2	-	-	-	1	-	4	9
Nausea and vomiting	-	-	-	-	-	-	-	-	-	
Neurosensory	1	-	-	-	-	-	-	-	1	2
Renal	-	-	-	-	-	-	-	-	-	
Infection	-	-	1	-	-	-	-	-	1	2
Early death	-		2		-		-		2	4
Toxic death	-		1		-		-		1	2
